# Comparative Effectiveness of Three Digital Interventions for Adults Seeking Psychiatric Services: A Randomized Clinical Trial

**DOI:** 10.1001/jamanetworkopen.2024.22115

**Published:** 2024-07-18

**Authors:** Adam G. Horwitz, Elizabeth D. Mills, Srijan Sen, Amy S. B. Bohnert

**Affiliations:** 1 Department of Psychiatry, University of Michigan Medical School, Ann Arbor; 2 Department of Anesthesiology, University of Michigan Medical School, Ann Arbor; 3 Molecular and Behavioral Neuroscience Institute, University of Michigan Medical School, Ann Arbor

## Abstract

**Importance:**

There is a substantial gap between demand for and availability of mental health services. Digital mental health interventions (DMHIs) are promising tools for bridging this gap, yet little is known about their comparative effectiveness.

**Objective:**

To assess whether patients randomized to a cognitive behavioral therapy (CBT)–based or mindfulness-based DMHI had greater improvements in mental health symptoms than patients randomized to the enhanced personalized feedback (EPF)–only DMHI.

**DESIGN,:**

**SETTING, AND PARTICIPANTS** This randomized clinical trial was conducted between May 13, 2020, and December 12, 2022, with follow-up at 6 weeks. Adult patients of outpatient psychiatry services across various clinics within the University of Michigan Health System with a scheduled or recent outpatient psychiatry appointment were recruited. Eligible patients were randomized to an intervention arm. All analyses followed the intent-to-treat principle.

**Interventions:**

Participants were randomized to 1 of 5 intervention arms: (1) EPF only; (2) Silvercloud only, a mobile application designed to deliver CBT strategies; (3) Silvercloud plus EPF; (4) Headspace only, a mobile application designed to train users in mindfulness practices; and (5) Headspace plus EPF.

**Main Outcomes and Measures:**

The primary outcome was change in depressive symptoms as measured by the Patient Health Questionnaire-9 (PHQ-9; score range: 0-27, with higher scores indicating greater depression symptoms). Secondary outcomes included changes in anxiety, suicidality, and substance use symptoms.

**Results:**

A total of 2079 participants (mean [SD] age, 36.8 [14.3] years; 1423 self-identified as women [68.4%]) completed the baseline survey. The baseline mean (SD) PHQ-9 score was 12.7 (6.4) and significantly decreased for all 5 intervention arms at 6 weeks (from −2.1 [95% CI, −2.6 to −1.7] to −2.9 [95% CI, −3.4 to −2.4]; n = 1885). The magnitude of change was not significantly different across the 5 arms (*F*_4,1879_ = 1.19; *P* = .31). Additionally, the groups did not differ in decrease in anxiety or substance use symptoms. However, the Headspace arms reported significantly greater improvements on a suicidality measure subscale compared with the Silvercloud arms (mean difference in mean change = 0.63; 95% CI, 0.20-1.06; *P* = .004).

**Conclusions and Relevance:**

This randomized clinical trial found decreases in depression and anxiety symptoms across all DMHIs and minimal evidence that specific applications were better than others. The findings suggest that DMHIs may provide support for patients during waiting list–related delays in care.

**Trial Registration:**

ClinicalTrials.gov Identifier: NCT04342494

## Key Points

**Question:**

What is the comparative effectiveness of mindfulness-based, cognitive behavioral therapy–based, or personalized feedback digital interventions in reducing mental health symptoms in adults seeking mental health treatment?

**Findings:**

In this randomized clinical trial of 2079 participants, depressive and anxiety symptoms improved across the intervention conditions during the 6-week trial. The magnitude of improvement did not significantly differ across various intervention arms.

**Meaning:**

Findings of this study suggest that different digital interventions can be used as supplemental or adjunctive tools within health care systems and may support patients during waiting list–related delays in care.

## Teaser

This randomized clinical trial assesses the comparative effectiveness of digital mindfulness-based, cognitive behavioral therapy–based, and personalized feedback tools for patients awaiting formal mental health treatment.

## Introduction

Mental health conditions are among the greatest contributors to disability and generate substantial societal costs in the US, affecting more than 1 in 5 people over their lifetime.^1,2,3^ While there are several moderately effective treatment options for common mental health conditions, including medications and psychotherapy, there is a wide gap between the demand and capacity for mental health services, prolonging wait times and increasing the prevalence of symptomatic adults with unmet mental health needs.^4,5^ Digital mental health interventions (DMHIs) are promising tools for bridging this gap between mental health care need and capacity.^6,7^

An estimated 85% of US adults own a smartphone, making DMHIs accessible to most of the population, including socioeconomically disadvantaged groups.^8^ In addition to increasing the capacity of the mental health care system, DMHIs are able to overcome common barriers to seeking mental health care, such as lack of time and concerns about privacy and cost.^9,10,11^ Meta-analyses across various DMHIs, particularly those incorporating skills from evidence-based treatments, such as cognitive behavioral therapy (CBT) and mindfulness, have reported improvement in depressive and anxiety symptoms with small to moderate effect sizes compared with control conditions.^12,13^ Furthermore, studies have demonstrated the benefits of personalized feedback–notification interventions that generate tips and strategies meant to support better health behaviors.^14,15^ Thus, although robust evidence has shown DMHIs to be effective compared with usual care or waiting list conditions, relatively little is known about the comparative effectiveness of different DMHIs.

To understand the potential role of DMHIs in bridging gaps in mental health care, in the present pragmatic randomized clinical trial (RCT), we evaluated the comparative effectiveness of different DMHIs for patients scheduled for in-clinic mental health visits. Given the interest in transdiagnostic approaches and the practicality of a universal approach to providing DMHIs to patients scheduled for outpatient services, intervention groups were evaluated for changes in depression, anxiety, substance use, and suicidality after 6 weeks of intervention. We hypothesized that (1) patients randomized to the passive enhanced personalized feedback (EPF)–only intervention would report less improvement in mental health symptoms compared with patients randomized to the more active CBT-based or mindfulness-based DMHIs and that (2) patients receiving both CBT-based or mindfulness-based DMHIs and EPF would show greater improvements than patients who received a CBT-based or mindfulness-based intervention alone without EPF. A secondary analysis compared the performance of the CBT-based and mindfulness-based interventions (regardless of EPF) across these symptom domains, but there were no a priori hypotheses regarding comparative effectiveness.

## Methods

This RCT was conducted from May 13, 2020, to December 12, 2022. Written informed consent was obtained from all participants. The University of Michigan Institutional Review Board approved all procedures in the study. The trial protocol is provided in Supplement 1. We followed the Consolidated Standards of Reporting Trials (CONSORT) reporting guideline.^16^

### Setting and Participants

Participants were adults seeking outpatient psychiatric services from several mental and behavioral health clinics within the University of Michigan Health System. Seventy-seven percent of patients were recruited from Michigan Medicine outpatient psychiatry clinics, with an additional 10% recruited through student-specific clinics and 13% from collaborative care occurring in primary care clinics. Included participants were English speakers aged 18 years or older with either a scheduled future mental health appointment or an initial appointment completed within the past 60 days and daily access to a smartphone for use in the study. Participants were excluded if they had cognitive or guardianship restrictions that prevented them from providing informed consent or if they had an active eating disorder that, based on patient or staff judgment, made the tracking device and activity monitoring contraindicated. Race and ethnicity data were collected merely to characterize the sample.

### Study Design and Procedures

Patients meeting initial inclusion criteria were contacted via telephone call, text, or email. Participants completed the baseline survey of demographics and clinical assessments using a health application (MyDataHelps; CareEvolution), received a study activity tracking device or used their own device (Fitbit; Google or Apple Watch; Apple), and linked their smartphone or wearable device to the MyDataHelps application.

Participants were randomized after completion of the baseline survey. The IQR for initial clinical appointments spanned from 5 days before to 40 days after study enrollment, with a third of participants completing a clinical appointment prior to formal enrollment. Participants received $20 for completing the baseline survey and another $20 for completing the 6-week follow-up survey. At prespecified intervals, study staff contacted participants by text or email if noncompliance with study configuration was identified and provided assistance to resolve technical problems. Reminders were also sent using notifications on the MyDataHelps application.

### Intervention Arms

There were 5 intervention arms: (1) EPF only; (2) Silvercloud (American Well Corporation) only, a mobile application designed to deliver CBT strategies; (3) Silvercloud plus EPF; (4) Headspace only, a mobile application designed to train users in mindfulness practices; and (5) Headspace plus EPF. Recommendations for daily use and specific modules or tracks (eg, depression or anxiety) were automatically provided for the Silvercloud and Headspace arms based on baseline symptoms.

Silvercloud has a global user base of over 500 000 people and includes psychoeducation and self-guided modules introducing CBT skills (eg, mood monitoring, activity scheduling, and cognitive restructuring) via text, videos, and journaling exercises. This mobile application has demonstrated considerable benefits of reducing depression and anxiety symptoms (effect size range, 0.50-0.63) compared with control conditions.^17,18,19^ Headspace includes a library of guided meditations and strategies for improving sleep, decreasing anxiety, and coping with stress. It has a user base of over 2 000 000 people and has also demonstrated efficacy in reducing depressive and anxiety symptoms (effect size range, 0.24-0.26) compared with control conditions.^20,21,22^

In the EPF arms, feedback was displayed on the MyDataHelps dashboard and delivered through pop-up notifications. A subset of notifications included personalized content based on their specific data, with content categories of physical activity, sleep, and mood. Example messages were “Giving to others can have the added bonus of making us feel good as well. Who can you do a small kindness for today? Notice the impact on your mood,” and “Your daily step average since the start of the study is [average daily step count]. Sometimes it’s tough to get moving, and yet you’ve had some really great active days. Keep it up!”^14^ Notification messages were delivered twice a day at 10 am and 2 pm to all participants in the EPF arm. Some of the messages for participants randomized to Silvercloud plus EPF or Headspace plus EPF were tailored to provide feedback and encouragement on using the application, with message categories such as therapy minutes for the Silvercloud plus EPF arm and mindful minutes for the Headspace plus EPF arm. The DMHIs were completely independent of the clinicians or health care system from which participants were awaiting services.

### Outcomes

The primary outcome of the study was change in depression score as measured by the 9-item Patient Health Questionnaire-9 (PHQ-9; score range: 0-27, with higher scores indicating greater depression symptoms).^23^ Secondary outcomes were changes in anxiety, substance use, and suicidality from baseline to 6-week follow-up. Anxiety was measured by the 7-item Generalized Anxiety Disorder-7 (GAD-7; score range: 0-21, with higher scores indicating greater anxiety symptoms).^24^ Substance use was measured using an adapted version of the Alcohol, Smoking, and Substance Involvement Screening Test (ASSIST)^25^ that rates frequency of use in the past 3 months for 9 different classes of substances on a Likert scale ranging from 0 (indicating never) to 4 (indicating daily or almost daily). Presence of any substance use was detected by the ASSIST outcome. Suicidality was assessed using the Positive and Negative Suicidal Ideation (PANSI), a 14-item measure with a negative subscale (PANSI–; score range: 8-40, with higher scores indicating greater risk for suicidal behavior) and a positive subscale (PANSI+; score range: 6-30, with lower scores indicating greater risk for suicidal behavior).^26^

### Randomization and Blinding

Participants were randomized using a 2:1:1:1:1 allocation, with the EPF-only arm being twice the size of other groups. Computer randomization was done in blocks of 12 and 18 with no stratification within MyDataHelps once the informed consent record was complete and patients were identified as eligible. The order was concealed from study staff who were enrolling patients; the study dashboard revealed a patient’s assignment when the patient completed all enrollment procedures and the baseline survey. Follow-up surveys were delivered via MyDataHelps, and outcomes were self-reported; therefore, no blinding procedures regarding outcomes assessment were necessary for study staff.

### Statistical Analysis

With primary analyses using the analysis of covariance (ANCOVA) test for intervention effects between the 5 groups, 95% power was achieved with a small effect size of 0.25 for a minimum sample size per group of 65, assuming 2 variables, a numerator *df* of 5 and α = .05, according to G*Power.^27^ Patient-specific change from baseline scores for the PHQ-9, GAD-7, and PANSI were calculated for all participants who completed the 6-week follow-up survey. All analyses followed the intent-to-treat principle. The effect of intervention arms on change from baseline for each of these outcomes was assessed through ANCOVA, adjusting for baseline value. The effect of study group on ASSIST at 6 weeks was assessed using logistic regression for the outcome of any substance use. ANCOVA was performed with PROC GLM in SAS, version 9.4 (SAS Institute Inc), with between-group comparisons using a Tukey-Kramer multiple-comparisons adjustment (which increases the *P* values of the between-group pairwise comparisons such that the global error rate for each outcome analyzed is *P* = .05). Two-sided *P* < .05 indicated statistical significance. The prespecified, secondary combined group comparisons used contrasts via ANCOVA for the full group. Logistic regression analyses were performed with PROC LOGISTIC in SAS, version 9.4.

## Results

### Study Participants

A total of 2079 participants completed the baseline survey (Figure). These patients had a mean (SD) age of 36.8 (14.3) years and self-identified as women (1423 [68.4%]), men (539 [25.9%]), or transgender or nonbinary (106 [5.1%]) and as American Indian or Alaska Native (8 [0.4%]), Asian (118 [5.7%]), Black (162 [7.8%]), Hispanic or Latino (142 [6.8%]), White (1590 [76.5%]), or multiple (99 [4.8%]) and other (78 [3.8%]) races and ethnicities (Table 1). Of these participants, 690 (33.2%) were randomized to the EPF-only arm, 345 (16.6%) to the Silvercloud-only arm, 346 (16.6%) to the Silvercloud plus EPF arm, 349 (16.8%) to the Headspace-only arm, and 349 (16.8%) to the Headspace plus EPF arm (Figure). Baseline demographics were similar between all study arms in terms of age, race, ethnicity, sex, gender identity, sexual orientation, employment status, and educational level (Table 1).

**Figure.**
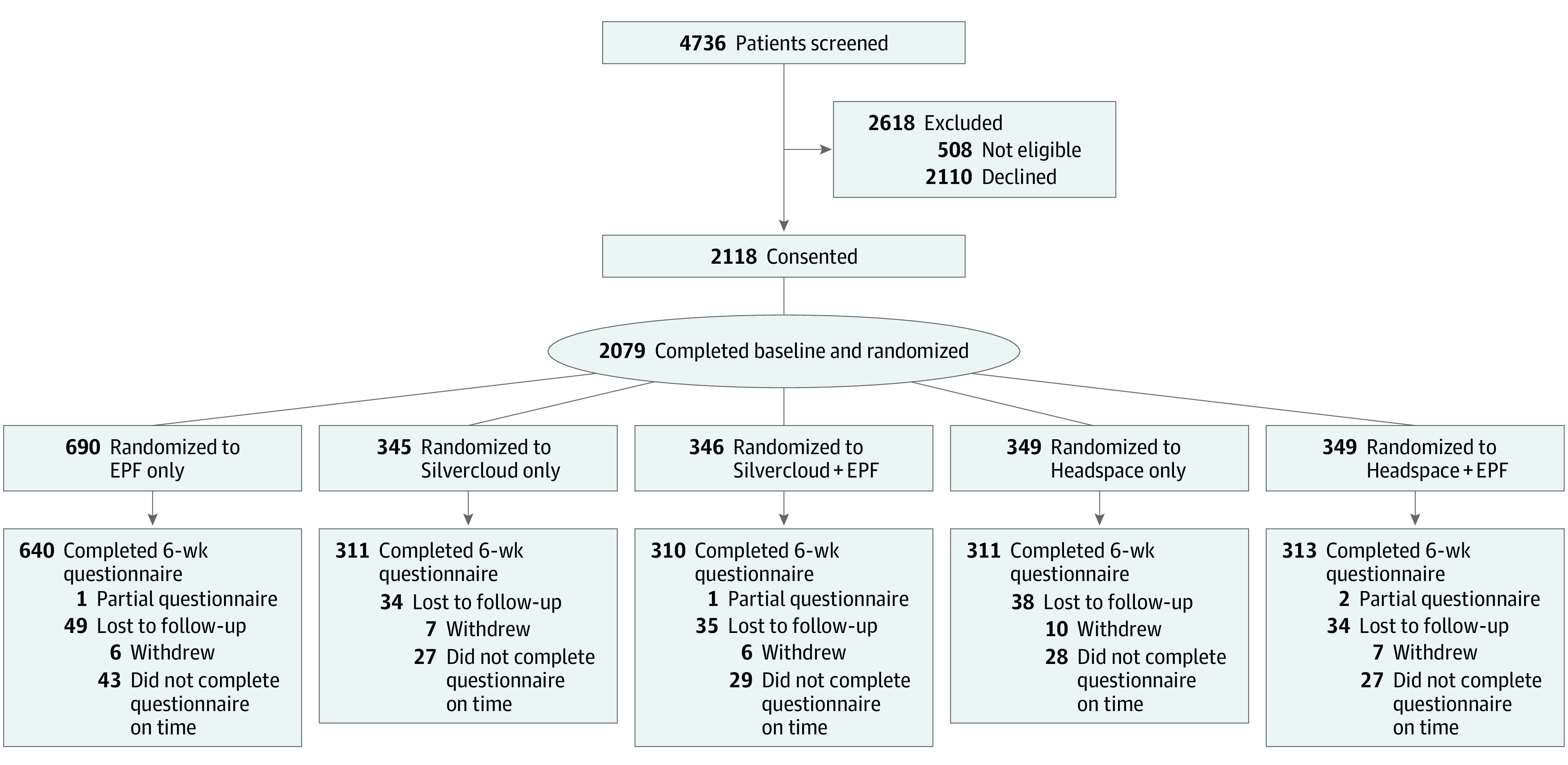
Flow Diagram of Study Participants EPF indicates enhanced personalized feedback.

**Table 1.** Participant Characteristics

Characteristic	Patients, No. (%)					
	EPF only (n = 690)	Headspace only (n = 349)	Headspace + EPF (n = 349)	Silvercloud only (n = 345)	Silvercloud + EPF (n = 346)	Total (N = 2079)
Age, mean (SD), y	37.3 (14.5)	36.2 (14.4)	36.8 (14.5)	36.8 (13.7)	36.5 (14.3)	36.8 (14.3)
Race^a^						
American Indian or Alaska Native	2 (0.3)	1 (0.3)	2 (0.6)	1 (0.3)	2 (0.6)	8 (0.4)
Asian	44 (6.4)	17 (4.9)	17 (4.9)	17 (4.9)	23 (6.7)	118 (5.7)
Black	57 (8.3)	33 (9.5)	23 (6.6)	31 (9.0)	18 (5.2)	162 (7.8)
White	516 (74.8)	269 (77.1)	275 (78.8)	259 (75.1)	271 (78.3)	1590 (76.5)
Multiple races	38 (5.5)	12 (3.4)	16 (4.6)	17 (4.9)	16 (4.6)	99 (4.8)
Other^b^	27 (3.9)	13 (3.7)	11 (3.2)	16 (4.6)	11 (3.2)	78 (3.8)
Unknown	6 (0.9)	4 (1.2)	5 (1.4)	4 (1.2)	5 (1.5)	24 (1.2)
Ethnicity^a^						
Hispanic or Latino	44 (6.4)	28 (8.0)	31 (8.9)	24 (7.0)	15 (4.3)	142 (6.8)
Sex						
Male	192 (27.8)	80 (22.9)	106 (30.4)	89 (25.8)	93 (26.9)	560 (27.0)
Female	491 (71.2)	267 (76.5)	242 (69.3)	251 (72.8)	248 (71.7)	1499 (72.1)
Unknown	7 (1.0)	2 (0.6)	1 (0.3)	5 (1.5)	5 (1.5)	20 (1.0)
Gender identity						
Man	192 (27.8)	78 (22.4)	97 (27.8)	84 (24.4)	88 (25.4)	539 (25.9)
Woman	466 (67.5)	248 (71.1)	231 (66.2)	242 (70.1)	236 (68.2)	1423 (68.4)
TGNB	28 (4.1)	19 (5.4)	21 (6.0)	18 (5.2)	20 (5.8)	106 (5.1)
Unknown	4 (0.6)	4 (1.2)	0	1 (0.3)	2 (0.6)	11 (0.5)
Sexual orientation						
Heterosexual	495 (71.7)	241 (69.1)	253 (72.5)	236 (68.4)	223 (64.5)	1448 (69.7)
Bisexual	91 (13.2)	53 (15.2)	44 (12.6)	50 (14.5)	63 (18.2)	301 (14.5)
Gay or lesbian	29 (4.2)	14 (4.0)	18 (5.2)	27 (7.8)	17 (4.9)	105 (5.1)
Other^c^	35 (5.1)	24 (6.9)	13 (3.7)	16 (4.6)	22 (6.4)	110 (5.3)
Multiple identities	20 (2.9)	8 (2.3)	10 (2.9)	8 (2.3)	10 (2.9)	56 (2.7)
Unknown	20 (2.9)	9 (2.6)	11 (3.2)	8 (2.3)	11 (3.2)	59 (2.8)
Employment status						
Full-time	261 (37.8)	136 (39.0)	150 (43.0)	141 (40.9)	137 (39.6)	825 (39.7)
Part-time	140 (20.3)	61 (17.5)	61 (17.5)	66 (19.1)	71 (20.5)	388 (19.2)
Retired	53 (7.7)	24 (6.9)	21 (6.0)	20 (5.8)	21 (6.1)	139 (6.7)
Student	91 (13.2)	53 (15.2)	42 (12.0)	45 (13.0)	47 (13.6)	278 (13.4)
Unemployed	143 (20.7)	75 (21.5)	75 (21.5)	72 (20.9)	70 (20.2)	435 (20.9)
Unknown	2 (0.3)	0	0	1 (0.3)	0	3 (0.1)
Educational level						
<High school diploma	14 (2.0)	7 (2.0)	12 (3.4)	6 (1.7)	9 (2.6)	49 (2.3)
High school diploma or GED	68 (9.9)	38 (10.9)	37 (10.6)	44 (12.8)	38 (11.0)	225 (10.8)
Some college	165 (23.9)	98 (28.1)	85 (24.4)	86 (24.9)	85 (24.6)	519 (25.0)
Associate degree or TVS	88 (12.8)	45 (12.9)	52 (14.9)	37 (10.8)	39 (11.3)	261 (12.6)
Bachelor’s degree	207 (30.0)	84 (24.1)	95 (27.2)	84 (24.4)	94 (27.2)	564 (27.1)
≥Master’s degree	146 (21.2)	77 (22.0)	68 (19.5)	87 (25.2)	80 (23.1)	458 (22.1)
Unknown	2 (0.3)	0	0	1 (0.3)	1 (0.3)	4 (0.2)

Abbreviations: EPF, enhanced personalized feedback; GED, General Educational Development; TGNB, transgender or nonbinary; TVS, trade or vocational school degree or certificate.

^a^ Race and ethnicity were self-identified.

^b^ Other race was self-identified as “something else.”

^c^ Other sexual orientation was self-identified as “something else.”

Participants’ age, race, and ethnicity were not significantly different from those of the clinic population from which they were recruited, but females were more likely to enroll (72.1% of the study sample and 65.6% in the clinic sample). This finding is consistent with many other studies reporting greater study participation among females than males.^28^

Most participants (1465 of 1885 [77.5%]) attended at least 1 clinical visit prior to taking the 6-week survey, including 595 (31.5%) attending 1 appointment, 307 (16.3%) attending 2 appointments, and 563 (29.8%) attending 3 or more appointments; there were no differences in clinical visits based on intervention arm. The median (IQR) time used for the 4 intervention arms that included Headspace or Silvercloud was 35.6 (127) minutes.

### Primary Outcome

The mean (SD) baseline PHQ-9 depression score was 12.7 (6.4). Overall, depression scores decreased by 2.5 points from baseline to 6-week follow-up (n = 1885) for all 5 arms (marginal mean differences [MDs] in mean change ranged from –2.1 [95% CI, −2.6 to −1.7] to –2.9 [95% CI, −3.4 to −2.4]); Table 2 provides within-group estimates and 95% CIs. The magnitude of change was not significantly different across the 5 intervention arms (*F*_4,1879_ = 1.19; *P* = .31) (eFigure 1 in Supplement 2) or when intervention arms were pooled based on (1) EPF only vs the 4 other arms (MD in mean change = –0.10; 95% CI, −0.51 to 0.32; *P* = .66), (2) Silvercloud plus EPF and Headspace plus EPF vs Silvercloud only and Headspace only arms (MD in mean change = –0.26; 95% CI, −0.75 to 0.22; *P* = .29), or (3) Silvercloud only and Silvercloud plus EPF vs Headspace only and Headspace plus EPF arms (MD in mean change = 0.46; 95% CI, −0.03 to 0.94; *P* = .07). Exploratory ANCOVA did not show significant differences in PHQ-9 symptom-level change based on attending 0, 1, or 2 or more appointments during the 6-week follow-up period. This finding suggests that in-clinic visits did not meaningfully contribute to symptom-level change across intervention groups.

**Table 2.** Symptom-Level Change for Depression Among the 5 Intervention Arms

	EPF only (n = 690)^a^	Headspace only (n = 349)^b^	Headspace + EPF (n = 349)^c^	Silvercloud only (n = 345)^b^	Silvercloud + EPF (n = 346)^d^
Baseline PHQ-9 score, mean (SD)	12.6 (6.4)	12.5 (6.3)	13.0 (6.8)	13.0 (6.2)	12.8 (6.4)
6-wk PHQ-9 score, mean (SD)	10.1 (5.8)	9.8 (5.9)	10.1 (5.9)	10.3 (5.9)	10.6 (5.7)
Change in PHQ-9 score, mean (95% CI)^e^	−2.4 (−2.8 to −2.1)	−2.9 (−3.4 to −2.4)	−2.7 (−3.1 to −2.2)	−2.5 (−2.9 to −2.0)	−2.1 (−2.6 to −1.7)

Abbreviations: EPF, enhanced personalized feedback; PHQ-9, Patient Health Questionnaire-9 (score range: 0-27, with higher scores indicating greater depression symptoms).

^a^ N = 640 for 6-week and change in values.

^b^ N = 311 for 6-week and change in values.

^c^ N = 313 for 6-week and change in values.

^d^ N = 310 for 6-week and change in values.

^e^ Symptom-level change estimates were adjusted for baseline PHQ-9 score via analysis of covariance. Mean changes in PHQ-9 scores were significantly different from 0 at *P* < .001 for all intervention arms. There were no significant differences in mean changes in PHQ-9 scores among the 5 intervention arms when adjusted for multiple comparison using a Tukey-Kramer adjustment (*F*_4,1879_ = 1.19; *P* = .31).

### Secondary Outcomes

Similar to PHQ-9 depression scores, GAD-7 anxiety scores significantly improved from baseline to the 6-week follow-up in all groups, and the magnitude of improvement was not significantly different across the 5 intervention arms in the 3 pooled comparisons. Table 3 provides within-group estimates and 95% CIs, and eFigure 2 in Supplement 2 provides boxplots.

**Table 3.** Symptom-Level Change for Secondary Clinical Outcomes Among the 5 Intervention Arms

Outcome	EPF only (n = 690)^a^	Headspace only (n = 349)^b^	Headspace + EPF (n = 349)^c^	Silvercloud only (n = 345)^b^	Silvercloud + EPF (n = 346)^d^
Baseline GAD-7 score, mean (SD)	11.5 (5.8)	11.6 (5.8)	11.4 (5.9)	11.9 (6.0)	11.8 (5.8)
6-wk GAD-7 score, mean (SD)	9.9 (5.9)	9.5 (5.4)	9.7 (5.7)	10.0 (5.6)	9.8 (5.7)
Change in GAD-7 score, mean (95% CI)^e^	−1.5 (−1.9 to −1.2)	−2.1 (−2.6 to −1.7)	−1.8 (−2.2 to −1.3)	−1.7 (−2.1 to −1.2)	−1.9 (−2.4 to −1.5)
Baseline PANSI+ score, mean (SD)	18.4 (5.0)	18.4 (5.1)	17.9 (5.1)	18.2 (4.9)	17.8 (5.3)
6-wk PANSI+ score, mean (SD)	19.1 (5.3)	19.6 (5.6)	19.0 (5.1)	18.8 (4.9)	18.2 (5.3)
Change in PANSI+ score, mean (95% CI)^e^	0.8 (0.5 to 1.1)	1.2 (0.7 to 1.6)	1.0 (0.6 to 1.4)	0.7 (0.2 to 1.1)	0.2 (−0.2 to 0.7)
Baseline PANSI– score, mean (SD)	11.1 (5.8)	11.0 (5.6)	12.2 (6.8)	11.6 (6.8)	11.7 (6.3)
6-wk PANSI– score, mean (SD)	9.9 (4.6)	9.9 (4.4)	10.8 (5.5)	10.5 (5.5)	10.6 (5.0)
Change in PANSI– score, mean (95% CI)^e^	−1.3 (−1.6 to −1.0)	−1.3 (−1.7 to −0.9)	−1.0 (−1.4 to −0.5)	−1.1 (−1.5 to −0.6)	−1.0 (−1.5 to −0.6)

Abbreviations: EPF, enhanced personalized feedback; GAD-7, Generalized Anxiety Disorder-7 (score range: 0-21, with higher scores indicating greater anxiety symptoms); PANSI, Positive and Negative Suicidal Ideation; PANSI+, positive PANSI subscale (score range: 6-30, with lower scores indicating greater risk for suicidal behavior); PANSI–, negative PANSI subscale (score range: 8-40, with higher scores indicating greater risk for suicidal behavior).

^a^ N = 641 for 6-week and change in values.

^b^ N = 311 for 6-week and change in values.

^c^ N = 313 for 6-week and change in values.

^d^ N = 311 for GAD-7 6-week and change in values; N = 310 for PANSI+ and PANSI– 6-week and change in values.

^e^ Mean change in value was adjusted for baseline scores via analysis of covariance (ANCOVA). Mean change in GAD-7 and PANSI– scores were significantly different from 0 at *P* < .001 for all intervention arms. For GAD-7 and PANSI– scores, there were no significant differences in mean change in scores among the 5 intervention arms. Mean change in PANSI+ scores were significantly different from 0 at *P* < .01 for all interventions except Silvercloud + EPF. Among the pairwise comparisons for PANSI+ scores, the only significant difference was Silvercloud + EPF vs Headspace only at 0.9 (95% CI, 0.1-1.8; *P* = .02). All pairwise comparisons were adjusted for multiple comparisons using the Tukey-Kramer method.

With respect to suicidality, on the PANSI+ subscale, the Headspace-only arm had significantly greater improvement than the Silvercloud plus EPF arm (MD in mean change = 0.94; 95% CI, 0.09-1.78; *P* = .02). There were no significant differences when arms were pooled based on EPF only vs the other 4 arms. However, pooled Headspace only and Headspace plus EPF arms had significantly greater improvement than pooled Silvercloud only and Silvercloud plus EPF arms (MD in mean change = 0.63; 95% CI, 0.20-1.06; *P* = .004). Scores on the PANSI– subscale decreased for all 5 intervention arms from baseline to follow-up, and the magnitude of improvement was not significantly different when examined individually or when pooled (Table 3). In sensitivity analyses, we examined outcomes after applying attrition weighting to address missing data at follow-up. Results did not reveal any significant differences in patterns of change for primary and secondary outcomes.

In logistic regression analyses, abstinence from substances did not significantly change during the study period overall and was not significantly different among any of the 5 intervention arms (abstinence ranged from 14.0% to 19.7% at baseline to 16.2% to 20.6% at follow-up; no individual arm had a more than 4.2% improvement in abstinence rates). Findings for change in substance use across groups were also not significant when alcohol and marijuana use were examined as individual, continuous dependent variables.

## Discussion

Contrary to our hypotheses, randomization to various DMHIs did not result in differing outcomes with respect to depression, anxiety, suicidality, and substance use among adults scheduled for outpatient psychiatry services. Specifically, there were no differences between participants randomized to receive passive EPF only compared with those randomized to an active commercial mobile application (Headspace or Silvercloud) with or without EPF. There were also no significant differences between patients who were randomized to Headspace only or Silvercloud only and those randomized to Headspace plus EPF or Silvercloud plus EPF. However, patients randomized to Headspace had significantly greater improvements on the PANSI+ subscale compared with those randomized to Silvercloud, although these improvements were negligible.

Participants across the DMHI groups had modest improvements in depression, anxiety, and suicide risk. The magnitude of change in depressive symptoms was similar to that in prior studies of mindfulness applications (SD change, 0.4),^21^ although it was slightly lower than prior CBT-based Silvercloud studies (PHQ-9 change range, 3.6-5.2).^17,18^ However, prior Silvercloud studies included clinician feedback and 15-minute reviews of application module use on a weekly basis, which was not included in the present study, and had more than 3 hours of median use time.^17,18^

Improvements in the current study may have been achieved with a human component and/or greater engagement. Most patients were already on a waiting list for more than 3 months before being contacted to schedule an initial appointment (and becoming eligible for the study), and symptom reduction was unrelated to the number of formal mental health visits, suggesting that the symptom reductions were not fully explained by alternatives, such as regression to the mean or parallel interventions. While this RCT was not designed to evaluate causality or efficacy, the improvements across intervention groups were consistent with prior literature demonstrating the effectiveness of these DMHIs^18,22^ and suggest that DMHIs were generally beneficial, without any intervention or pairing of interventions being notably superior to the others evaluated in this study.

### Strengths and Limitations

This study has several strengths, including a large transdiagnostic clinical population of treatment-seeking adults and a pragmatic design embedded in existing service delivery health care systems. However, the findings must be considered within the context of the existing limitations of the study. The benefits of the pragmatic design come at the expense of greater control over facets of enrollment, including wait time for services and other potential confounders. Most participants attended formal psychiatric care visits within the 6-week study period. Although a sensitivity analysis did not find significant differences when adjusting for number of clinical visits, there may have been aspects of formal care that contributed to the improvement in symptoms across groups and diluted the ability to identify differences between applications in effects on symptom reduction. The comparative effectiveness design with no usual care control arm was chosen to increase acceptability by participants and to maximize enrollment, yet this design did not allow us to conclude definitively whether symptom reductions resulted from the interventions, although efficacy has been previously well demonstrated.^17,18,19,20,21,22^ The study sample was diverse with respect to including various presenting clinical concerns at various clinics but had similar demographic characteristics to those of the treatment-seeking population (ie, mostly White and female patients), and it is unclear whether findings may be generalizable to more diverse samples. While differences were not observed in intervention effects on depression, anxiety, suicidality, or substance use outcomes at the 6-week follow-up, there may have been other unmeasured, clinically relevant factors or mechanisms that responded differently as a function of randomization to the DMHIs.

## Conclusions

This RCT compared the effectiveness of several DMHIs for patients awaiting mental health care. Depression and anxiety symptoms decreased consistently across all interventions, and there was minimal evidence that specific applications were generally better than others. The findings highlight the potential for DMHIs to be offered as supplemental or adjunctive tools within health care systems. While the modest magnitude of improvement does not suggest DMHIs can replace formal mental health care services, DMHIs may serve an important role in supporting patients during waiting list–related delays in care.

## Notes

Supplement 1.
Trial Protocol

Supplement 2. **eFigure 1.** Symptom-Level Change in Depressive Symptoms Across Intervention Arms **eFigure 2.** Symptom-Level Change in Anxiety and Suicidality Symptoms Across Intervention Arms

Supplement 3.
Data Sharing Statement
