# Supplementary material for: Comparative Effectiveness of Three Digital Interventions for Adults Seeking Psychiatric Services: A Randomized Clinical Trial
Source: JAMA Netw Open. 2024 Jul 18;7(7):e2422115. doi: 10.1001/jamanetworkopen.2024.22115 (PMC11258584; doi:10.1001/jamanetworkopen.2024.22115)
Supplement: Supplement 1. — Trial Protocol [file jamanetwopen-e2422115-s001.pdf]

PROTOCOL

Version 8
Last Updated: 3/19/2021

**Study Title:**	**Providing Mental Health Precision Treatment (PROMPT)**
**Full Study Title:**	Enhancing Mental Health Care Through Mobile Technology
**Principal Investigators:**	Amy Bohnert, PhD<br>Srijan Sen, MD, PhD

PROBLEM STATEMENT

Depression, sleep concerns, addiction, and anxiety are leading and growing causes of disability, productivity loss, and premature mortality globally. The number of behavioral health clinicians available to provide face-to-face, traditional care is woefully inadequate to meet the growing need. Further, a substantial proportion of patients who receive traditional care do not get better. With inadequate evidence to meaningfully guide treatment decisions and few available objective measures of mental health symptoms, choice of treatment is often based on clinician preference and simple heuristics.

More than any other advance that has emerged in the past four decades, mobile technology has the potential to address the dual problems of limited clinical capacity and inadequate and untimely data. Mobile technology holds the potential to both track and intervene on mental health symptoms in powerful ways that had not previously been possible. However, little is known about how to derive the greatest value from this technology by targeting patients most likely to benefit and by providing clinicians with the most useful information gleaned from the intensive data collection processes in order to inform treatment decisions. Additionally, investigators continue to analyze genetic information as a predictor for mental health symptoms and outcomes. Genetic analysis, especially when coinciding with data gathered from mobile technology, may have the capacity to even further inform mental health treatment decisions and allow for comprehensive precision treatment.

OBJECTIVES

As part of the Precision Health initiative at the University of Michigan, this project is focused on the effects of wearable and mobile technology to both reduce mental health symptoms and predict response to clinic-based treatments.

The overall goal of this project is to reduce the burden of mental health conditions by two means:

1) Increasing capacity in the mental health care system through expanding use of mobile technology-delivered interventions.
2) Accelerating recovery by matching patients to the pharmacological, psychological, and mobile-based treatments from which they will likely derive the greatest benefit.

To do so, we will complete the following **specific aims**:

Aim 1: To assess the independent and combined effectiveness of two classes of mobile technology interventions among patients while on the waitlist for traditional care:
(a) App-based cognitive behavioral and mindfulness interventions
(b) Enhanced objective, mobile data feedback on mood, sleep, heart rate, and activity levels

Aim 2: To use machine learning to identify key predictors of treatment response from mobile technology,

genomic, and environmental data collected from patients receiving:
(a) Mobile technology interventions while on a waitlist for outpatient mental health care
(b) Traditional face-to-face psychotherapy, medication, or neuromodulation treatment

RESEARCH DESIGN AND METHODS

This study will have **two phases**, each with different study designs:

> **Phase 1** will cover the period while individuals are waiting for care. The design of this phase is a randomized clinical trial, wherein participants are randomly assigned to one of three study conditions that vary by the type of Mobile Health (mHealth) intervention they can receive.
>
> **Phase 2** begins when a participant has a first appointment for outpatient mental health care, and uses a cohort study design for measuring outcomes associated with clinic-based treatments.

Phase 1

Patients with scheduled intake mental health appointments, pre-screening mental health appointments, on the waitlist/referral list, or identified as a potential patient seeking mental health care at Michigan Medicine or University Health Services will be recruited to participate in the study. Patients can be recruited at any time between the appointment being scheduled to up to one month following their intake appointment.

Participants that are enrolled with sufficient time before their scheduled appointment (≥ 2 weeks) will be randomized to one of **three groups**:

1) Enhanced Feedback + Standard Feedback (**EF+SF**): Participants will receive enhanced feedback from the MyDataHelps study app in addition to standard feedback from the Apple Watch health app or Fitbit activity tracker
2) App-Based Intervention + Standard Feedback (**App+SF**): Participants will receive an app-based intervention in addition to standard feedback from the Apple Watch health app or Fitbit activity tracker
3) App-Based Intervention + Enhanced Feedback + Standard Feedback (**App+EF+SF**): Participants will receive an app-based intervention, enhanced feedback from the MyDataHelps study app, and standard feedback from the Apple Watch health app or Fitbit activity tracker

Some patients may have a shorter waiting period (<2 weeks) prior to their intake appointment (e.g., due to acute mental health symptoms). These patients will also be contacted for recruitment to provide opportunity for benefit from the research and from access to the mHealth interventions and will still be randomized to one of the above-listed groups.

As part of the enrollment process, participants will be guided by a study team member through **three processes**:

1. **Download Study App:** Participants will download the study app, MyDataHelps, on their smartphone. MyDataHelps is a commercially available application developed by CareEvolution. This app serves as a vehicle for consent, data collection, delivery of all study questionnaires, automated notifications, and reminders. The app will also receive data from the Health app and study devices. Participants will be asked to complete daily mental health symptom assessments via the app throughout the duration of their enrollment in the study. The app will also present enhanced feedback on participant progress via a personalized “dashboard.” If participants are utilizing an Apple Watch with their existing iPhone, they must enable MyDataHelps to read out the data fields in the Health app. This will be made clear to

participants during the consent process, so a participant can decide if they are still interested in participating.

2. **Receive Device:** Participants will be provided with an Apple Watch or Fitbit to sync with their smartphone. Participants with iPhones will be provided with Apple Watches or Fitbits and participants with Androids will be provided with Fitbits, or similar smart watches and activity trackers for both types of phones. This device will be worn throughout their entire involvement in the study. This will allow for collection of objective data on daily activity, such as steps/physical activity, sleep, and heart rate.
3. **Download App-Based Intervention:** Participants randomized to the groups receiving app-based interventions will download additional apps to their smartphones that are designed to address mental health symptoms. Participants will be computer-assigned to one of the two app-based interventions (mindfulness via the Headspace app or cognitive behavioral therapy via the SilverCloud app). A study team member will guide them through the process of downloading the app to their phone at that time.

Participants will also be asked for permission for the study team to collect active (e.g., symptom self-reports) and passive (e.g., screen time) data through their smartphone through the duration of the study.

Surveys
Participants will be asked to complete four surveys, including a baseline survey at the time of enrollment and three follow-up surveys, which will be designed to complement in-clinic assessments completed as a part of standard outpatient mental health care. To the extent possible, redundancy with in-clinic assessments (i.e., due to the Measurement-Based Care program) will be avoided. The first follow-up survey will occur at the end of 6 weeks post-enrollment, the second at the end of 18 weeks post-enrollment and the third at the end of 12 months post-enrollment.

Depending on waitlist length for outpatient mental health services, there may be variation in whether participants have engaged with clinical care by the time they complete their follow-up survey(s). Surveys will be administered by the predetermined schedule regardless of whether participants have engaged with care. The rationale for this decision, rather than scheduling follow-up surveys to coincide with the initiation of outpatient care:

1. There is a possibility that participants may never engage with outpatient care, and these non-engagers would never receive follow-up surveys
2. Waitlist time often varies between recruitment sites (i.e., UHS tends to have a shorter waitlist for care), which would create measurement biases (e.g., UHS patients may consistently engage in care with less time to engage with the app-based intervention)
3. Participants may be receiving other mental health care (e.g., via primary care), making it less important to time follow-up surveys with the initiation of outpatient mental health services
4. The existing timeline ensures all participants randomized to the groups receiving app-based interventions have received a reasonable ‘dose’ of the intervention prior to assessment
5. The existing timeline ensures that measurement will take place at generally the same time frame for all participants

Summary of tasks that participants will be asked to complete during Phase 1:

- Complete daily mental health symptom assessments via the MyDataHelps app
- Wear assigned device (Apple Watch or Fitbit) regularly for objective data collection
- Engage with assigned app-based intervention (Headspace or SilverCloud) regularly
- Complete baseline assessment via the MyDataHelps app
- Complete 6-week follow-up survey

- Complete 18-week follow-up survey
- Complete 12-month follow-up survey
- Provide a biological specimen

<u>Phase 2</u>

Phase 2 begins when participants establish care with a clinical provider at Michigan Medicine Outpatient Psychiatry or UHS. In Phase 2, participants will engage with outpatient mental health care services as part of routine care, and the study will continue to gather both active and passive data on participants' symptoms and activity, similar to Phase 1. Participants will continue to have access to their assigned app-based intervention while concurrently receiving outpatient mental health services. The study will collect data from participants' phones and apps for up to one year following the initiation of clinical care or until participants have completed their 12-month follow-up survey. Participants may still remain enrolled in the study if they cease engagement with outpatient mental health care services during this phase.

Summary of tasks that participants will be asked to complete during Phase 2:

- Complete daily mental health symptom assessments via the MyDataHelps app
- Wear assigned device (Apple Watch or Fitbit) regularly for objective data collection
- Engage with assigned app-based intervention (Headspace or SilverCloud)
- Complete 6-week follow-up survey
- Complete 18-week follow-up survey
- Complete 12-month follow-up survey
- Provide a biological specimen (if not yet completed during Phase 1)

Screening and Recruitment

Participant screening and recruitment will be managed by study staff. All study team members are required to complete team/study training and all appropriate regulatory training and disclosures.

The study will recruit 4,500 participants via patients at Michigan Medicine and student patients at University Health Service (UHS). Potential study participants will include adult patients (age 18+) that are scheduled for an intake mental health appointment, pre-screening mental health appointment, on the waitlist/referral list, or identified as a potential patient seeking mental health at either Michigan Medicine or UHS. These appointments are generally scheduled with a 3-10 week waiting period. Patients will be invited to participate in the study as soon as possible after scheduling their appointment in order to best maximize participation during their full waiting period.

A waiver of HIPAA authorization is requested in order for study staff to:

1) Receive daily lists of patients for whom a mental health intake appointment has been scheduled at each recruitment site
2) Access the Michigan Medicine and UHS electronic health records to confirm eligibility of listed patients and obtain contact information

Study staff will employ the following **recruitment strategies** to contact, screen, and enroll potential participants:

1. At the time of scheduling their intake mental health appointment, patients may be informed by call center staff that they may be contacted about a new study.

2. Daily lists of newly scheduled appointments and individuals on the waitlist/referral list will be generated by the Michigan Medicine Department of Psychiatry call center, UHS scheduling, or from the Research Data Warehouse (RDW) and provided to study staff.
3. Using existing Research Data Warehouse information, a patient is prospectively identified as someone who meets the study criteria during the screening process. Potentially eligible patients may be contacted by email/text message/phone call to introduce the study, with an invitation to contact the study team by phone or email if interested in participating.
4. Prior to contacting patients by phone/text message/phone call, study staff will complete preliminary screenings via listed patients' electronic health records to determine whether basic, objective inclusion criteria are met (e.g., scheduled outpatient mental health appointment, age 18+).
5. Study staff will follow up with a phone call/text message/phone call within 2 business days of the patient's appointment scheduling to further describe the study and assess interest.
6. Patients identified by preliminary screenings as potentially eligible may be sent a standardized study letter by mail to introduce the study, with an invitation to contact the study team by phone/text message/email if interested in participating.
7. Interested patients will be asked additional screening questions to determine eligibility (e.g., owning a smartphone, willingness to keep study setup configured on smartphone for duration of study). Eligibility screening questions may be administered via phone contact with the potential participant or within the MyDataHelps app prior to consent.
8. Interested and eligible patients will be invited to the study via the RKStudio study portal email system and directed to download the MyDataHelps study app on their smartphone. Account creation within the app will facilitate participant access to the informed consent document and enrollment in the study.

Patients that have a short waiting period (<2 weeks) prior to their intake appointment will be offered the option to meet a study team member in person at the time of their clinic intake appointment for screening, consent, and enrollment. Study staff will conduct a preliminary screening via the electronic health record for eligibility, and will then contact potentially eligible participants by phone or email to offer a meeting at their first appointment to complete the study enrollment processes. If the patient does not show for the intake appointment, study staff will follow up by phone to offer further information and screening for the study. These participants may also opt to complete the study processes remotely (e.g., phone, app) or in a separate agreed-upon location (e.g., in the community), if desired.

Consent

Informed consent will be conducted within the MyDataHelps app via an eConsent. A waiver of documentation of consent is requested to obtain electronic consent for this study.

If a patient is interested in participating, a study team member will invite them to the study using the RKStudio study portal email system. Participants will be directed to download the MyDataHelps app on their smartphone through the Apple or Google Play Store (dependent on smartphone), which will include an IRB-approved study consent module. When a participant opens MyDataHelps, they are first required to register an account using their email, and are subsequently presented with the eConsent form that lays out the process of participation. The patient is asked their name and for a finger-drawn signature. Study personnel will be available by phone or email to answer any questions participants may have about the consent and study. If participants do not complete the informed consent process via the study app and have not indicated that they do not wish to participate in the study, study staff will follow up by phone or email to answer questions or provide assistance.

Participants will have the option to download a copy of the consent document or have a copy emailed or mailed to them.

Enrollment

Participants will complete several steps prior to enrollment being considered as "completed."

Participants must complete the following steps to be enrolled in the study:

1. MyDataHelps study app download, installation, and account creation
2. Completion of informed consent
3. Completion of baseline survey via the MyDataHelps study app
4. Download and installation of assigned app-based intervention (Headspace or SilverCloud), if applicable
5. Receipt of device (Apple Watch or Fitbit) and agreement to wear the device regularly. Participants' devices will be mailed, unless they indicated that they would like to meet with study staff to receive their device in person.
6. Pairing and setup of smartphone, device (Apple Watch or Fitbit), and MyDataHelps study app

Study staff are available by phone or email as necessary to provide technical support to aid participants in downloading apps, completing informed consent, or setting up their devices. Participants may also opt to meet with study staff in person, as described above.

Participants will be offered to complete consenting and enrollment steps remotely (via telephone with a study team member and on their own following their download of the MyDataHelps study app). If further assistance is needed, participants may opt to meet recruitment staff in person for support with downloading app(s), completing the informed consent process, completing the baseline survey, or picking up and configuring their Apple Watch or Fitbit. Patients who begin the recruitment process online may also opt to meet recruitment staff in person to complete the remainder of the process (e.g., informed consent, enrollment, downloading apps). In-person meetings may take place in a designated study space at Michigan Medicine or UHS or at an agreed-upon location in the community (e.g., coffee shop, campus library).

Inclusion Criteria

1. Must have a scheduled intake mental health appointment, pre-screening mental health appointment, on the waitlist/referral list, or identified as a potential patient seeking mental health care at Michigan Medicine or University Health Services
2. Age $\geq$ 18 years
3. Must have daily access to a smartphone to use for the study (iPhone 5s or later, depending on Apple Watch version available, or Android smartphone compatible with relevant Fitbit model and study app)
4. Understands English to enable consent and use of the MyDataHelps app and app-based interventions
5. Provide complete, updated contact information upon enrollment to the study
6. Agree to be contacted by study staff during the study
7. Willingness to keep study setup (devices, apps, settings) in required configuration for the duration of the study, and following instructions by study staff if required to update or re-achieve required configuration if required configuration has been lost (e.g., change of phone, app deletion)

Exclusion Criteria

1. Deemed unable to provide informed consent (e.g., cognitive inability, guardianship)
2. Eating disorder, unless well controlled, as self-reported by the patient, verified by the research participant's mental health team, or review of medical chart by study team
3. Scheduled outpatient mental health appointment is a pediatric appointment (even if age 18+)
4. Wrist too large or too small to wear an Apple Watch or Fitbit comfortably (Note: bands that are shorter or longer than the standard will be made available to minimize this exclusion)

Those who have known allergies or previous reactions to fluorocarbon-based synthetic rubber, such as contact dermatitis with fluoroelastomer bands primarily used in wrist-worn fitness devices, will be offered an alternate Apple Watch or Fitbit band.

Participants may be excluded at the discretion of the Principal Investigators based on the determination that it is in the participant’s or study staff’s best interest (e.g., to fully ensure participant privacy in the case that a study staff member knows a patient or their family personally).

Participant Remuneration

Participants will be remunerated $20 for completing the baseline survey, $20 for the first follow-up survey, $40 for the second follow-up survey, and $50 for the third follow-up survey. Participants could receive a total of $130 for surveys via cash or gift card if they complete all study activities. Participants will be given an Apple Watch or Fitbit to use during their participation in the study and may keep these devices once they have completed the study. The Apple Watch Series 3 is valued at about $250 and Fitbit Charge 3/Inspire HR valued at about $120. Other versions of these devices or similar devices may be given to participants, based on availability. Between completion of surveys and receipt of Apple Watch, Fitbit, or similar devices, total participant remuneration will range from $250 to $380 via cash or gift cards and receipt of mobile health device, dependent on the mobile health devices available and received.

Required Device Configuration

Participants will be guided through the study setup configuration requirements during the enrollment process. For the full study duration, participants will maintain the basic study configuration on their study devices (e.g., MyDataHelps, app-based intervention, Apple Watch or Fitbit synced with smartphone). Study staff will reach out to participants if non-compliance with study configuration is noted and will offer re-education or help to resolve technical problems. In interactions with participants, study staff will remind participants to ensure their devices are compliant with required device configuration. Notifications in MyDataHelps will support the participant in maintaining the required device configuration by sending update reminders.

A small minority of participants may experience study hardware or software problems. We may contact these participants to troubleshoot the problems via phone, letter, or email. An in-person meeting with study staff will be offered for further troubleshooting, if needed. If study tasks are completed successfully, these issues will have no effect on their compensation. However, if participants are unable to complete study tasks (e.g., follow-up surveys) due to these issues, their compensation may be impacted.

ASSESSMENT METHODS AND MEASURES

This study will combine the following sources of information about participants:

1) Self-report surveys
2) Michigan Medicine and UHS electronic health records and other medical records data
3) Biological specimen

4) Passive and active data collected from participants' phones, linked sensors (Apple Watch or Fitbit), or use of app-based interventions (SilverCloud or Headspace)

Self-Report Surveys

Participants will complete four surveys, including a baseline survey and three follow-up surveys. All surveys will be self-administered via the MyDataHelps study app. In the case of technological difficulties, the study team does have paper copies of the baseline and follow-up surveys available, and the surveys may be administered in person or by phone. The baseline survey will take about 25 minutes. Survey questions include demographics, overall physical and mental health symptoms and diagnoses, substance use, environment, use of mobile technology and health apps, and social network and support. For each of the three follow-up assessments, participants will complete an approximate 20-minute survey with similar content as the baseline survey. If necessary, the survey can be administered by study staff via phone who will read survey questions to the participant and record their responses. When participants are due for their surveys, they will receive an email or app notification that their survey is available via the MyDataHelps app. If participants do not respond to the questionnaires, and have not indicated that they do not wish to participate in the study, a reminder will be sent by email, phone, mail, or text message.

The first follow-up survey will occur at the end of 6 weeks post-enrollment, the second at the end of 18 weeks post-enrollment, and the third at the end of 12 months post-enrollment. Each of these time-points may occur near the time of an in-person outpatient mental health clinic appointment, at which participants may be asked to answer some of the same questions that are contained in the study surveys. To reduce participant burden, study staff will monitor upcoming appointments and participants will receive a version of the survey without redundant items if they have an appointment within 1 week of their follow-up survey due date. They will still complete the remainder of the follow-up survey and be paid the same amount for their time. Additionally, participants who do not show for clinic appointments will still be able to complete the follow-up assessments via standard study procedure.

Measures

Baseline and follow-up self-administered measures are reliable and valid among adults. Data will be collected via the MyDataHelps app, online via Qualtrics, or by phone with study staff. Please refer to Table 1 for a full list of study measures and administration time points.

Daily mental health symptom assessments will be collected via the MyDataHelps app. During app set-up, participants will be prompted to allow the app to send them notifications; study staff will encourage participants to enable notifications. A notification will be automatically generated on their smartphone on a daily basis and will appear as follows: "On a scale of 1 to 10, what was your average mood today?" Participants will then respond in the MyDataHelps app. Participants will be asked to complete this daily, one-item assessment throughout the duration of their study involvement.

Participants may also be asked to complete a weekly suicide risk assessment question via the MyDataHelps app. Participants would receive a notification to complete the weekly assessment within the app. The question will appear as: "On a scale from 0 (Not at all confident) to 10 (Extremely confident), how confident are you that you will NOT attempt suicide in the future?" (Czyz, et al., 2016).

Participants will also be asked about their frequency of use of mental health, physical health, and mindfulness apps via the MyDataHelps app. This assessment will take place as part of the baseline and all follow-up surveys. The questions are adapted from mental health app survey questions developed by Kern, et al., 2018, and will appear as follows:

- How frequently did you use mental health apps within the past 6 months? (Never, Less than monthly, Monthly, Weekly, Daily or almost daily)
- How frequently did you use physical health apps within the past 6 months? (Never, Less than monthly, Monthly, Weekly, Daily or almost daily)
- How frequently did you use mindfulness or meditation apps within the past 6 months? (Never, Less than monthly, Monthly, Weekly, Daily or almost daily)

Electronic Health Record Data

Participation in the study will allow for the use of the patients' electronic health record (EHR) data, outcomes registries (including local, state, national, and international, such as the National Death Index), prescription-related data accessed via the SureScripts Health Information Network, administrative data, and other sources of health and outcomes data stored in the University of Michigan Research Data Warehouse. Patients agree to be re-contacted in the event that researchers would like to consent the patients for additional studies requiring further testing, data, or feedback. They may decline any further study opportunities without any impact on their participation or compensation for this study.

Biological Specimen

A biological specimen will be obtained as part of this study, which will be facilitated by the University of Michigan Medical School Central Biorepository (CBR). The CBR will facilitate ordering of DNA collection kits, as well as the processing and storage of DNA samples. The Michigan Genomics Initiative (MGI) (HUM00071298) is a collaborator in this study, and MGI will cover the costs of DNA collection kits, processing, and storage that are charged by the CBR. Participants that have previously participated in MGI will not receive genetic analyses as part of this protocol, given that their biological specimen has already been submitted to the CBR.

Participants will be sent DNA collection kits via postal mail and asked to provide a small sample of saliva. Participants will return samples to study team members in a provided self-addressed, stamped return envelope. Participants also have the option to provide a saliva sample in person, if needed or preferred.

Genetic analyses will be carried out on the DNA sample. Incidental findings from the DNA analysis will not be shared with the participant or the participant's physician.

If participants do not submit a DNA sample and have not indicated that they do not wish to participate in the study, they will be contacted by a study team member by phone, email, text message, or mail as a reminder. Study staff will be trained to discuss confidentiality and privacy of genetic data to improve participant adherence with the request to submit a DNA sample.

Data Collected from Phones and Sensors

Data that may be collected from participant smartphones and periodically transmitted to a secure database may include:

- Phone orientation
- Phone acceleration
- Frequent locations
- Voice
- Social network indicators (call and text frequency, etc.)
- Screen time
- App usage

Words typed are stripped from their context and syntax, thus preventing the content of any messages from being deciphered. The study app does not collect phone numbers or email addresses, photos or videos, passwords, credit card numbers, or any other secure or personally identifying information.

Wearable device (Fitbit or Apple Watch) costs will be covered by the Precision Health award budget (devices will not be provided for free by Apple or Fitbit). Data collected from the Apple Watch or Fitbit include:

- Heart rate
- Physical activity (steps, minutes, type of activity, estimated calories)
- Geospatial location
- Sleep

Data collected from participant use of SilverCloud or Headspace will include general usage data, such as time spent utilizing the app per day.

MHEALTH INTERVENTIONS

Standard Feedback (SF)

All participants will receive the feedback that is standard with the Apple Watch or Fitbit devices. Both sensors provide feedback on activity level, heart rate, and progress toward daily activity goals on the device itself. For the Apple Watch, this also includes feedback from the iPhone's "Activity" and "Watch" apps. For Fitbit users, this includes feedback in the "Fitbit" app.

Enhanced Feedback (EF)

Participants randomized to receive enhanced feedback will receive this feedback from the MyDataHelps study app. This includes varying types of text and visual feedback based on data collected through the app. Feedback will be displayed to participants on a dashboard in the app or delivered via pop-up notifications. Participants will receive a combination of text and visual feedback.

Examples of potential text feedback:

- A summary statement connecting behaviors (e.g. "On Tuesday, your mood was a 9. That night, you slept 6 hours. Your mood was a 7 the next day.")
- Empathetic feedback about behavioral patterns (e.g. "You had a tough week. On Tuesday, when you were at the hospital for 12 hours, your mood was a 6. What have you done in the past to help your mood when you had a hard day?")
- A motivational tip (e.g. "It seems like you have had a tough week. Hang in there – you've survived worse!")

Examples of potential visual feedback:

- A graph plotting one data type over time (e.g. hours of sleep for the past week)
- A graph plotting the relationship between different data types over time (e.g. mood and sleep hours for the past month)

We also plan for some feedback to be customized based on subjects' mood, activity, and sleep data. For example, subjects who slept for less than 6 hours on average in the past week might receive different feedback than those who slept more.

App-Based Interventions

Participants randomized to the groups receiving the apps will be asked to install an mHealth app onto their smartphone. The two apps that will be used are Headspace, an app designed to train the user in mindfulness practices, and Silvercloud, an app designed to deliver cognitive behavioral therapy. These apps were selected because of evidence of efficacy and applicability across a wide range of mental health symptoms. Participants receiving apps will be computer-assigned to receive one of the two options. Participants who were recruited with <2 weeks prior to their scheduled appointment (and thus not included in the initial randomization) will receive access to one of the two apps at the time of their intake appointment, also based on computer random assignment.

App notifications will encourage participants to use the apps regularly. If user data indicates that a participant is not using the apps, study staff will contact the participant via app notification, phone call, email, letter, or text message. SilverCloud allows for integration of a health coach as a supportive role for increasing engagement, largely through asynchronous messaging. Study staff will be trained in the role of the health coach and will engage with participants through the app to promote app use, personalization, and overall engagement. For both apps, study staff will provide ongoing technical assistance and address any participant barriers to app use.

ANALYSIS PLAN

A primary goal is to obtain information about the feasibility and acceptability of the use of app-based interventions and enhanced feedback in response to mobile health device use, as well as to estimate the distribution and variability of potential primary and secondary outcomes of interest. Data will be examined using standard univariate summary measures, bivariate statistical tests of association, and graphical displays. We will assess subjective and objective data in areas including mental health and physical health measures, substance use, and app use. This will assess responsiveness to the interventions as well as how best to provide clinicians with useful patient data that may help inform treatment decisions. This is a primary outcome for future work. Additional outcomes to be assessed are listed in Table 1.

We will calculate means and standard deviations for participant characteristics and outcomes measures at each assessment and also for change scores. With outcomes that are categorical, we will use the Chi square test, and with outcomes that are continuous, we will use t-tests to make comparisons.

We will also conduct baseline and ongoing analyses of the presence of genotype x stress interaction among this sample. For example, as a baseline analysis, we will investigate association between each variant/haplotype and PHQ (baseline) scores using linear regression. To explore gene x environment interactions, we will assess for association between each variant/haplotype and PHQ (change) scores.

POTENTIAL RISKS, MINIMIZING RISKS, AND POTENTIAL BENEFITS

Potential Risks and Minimizing Risks

Breach of confidentiality (Rare likelihood of risk)
Several measures have been taken to reduce the risk of breach of confidentiality. These include training of study team members, electronic and physical security measures for data capture and storage, and collecting a minimum of identifiable information for each individual participant. The study will also pursue a Certificate of Confidentiality, pending IRB approval. Unique identification (ID) numbers will be assigned to all participants who complete the assessments. All data forms and assessments will be coded with this number rather than with participants' names. Breach of confidentiality will be considered a "definitely related" Serious Adverse Event. As such, it will be reported to the University of Michigan IRB within 7 days of occurrence, and a remediation plan will be put in place immediately.

Collection and storage of biospecimens (Rare likelihood of risk)
Saliva collection kits will be mailed to participants who will self-collect saliva by spitting in a coded tube and returning in a pre-addressed, stamped envelope with no participant identifiers. DNA will be stored in a coded manner for future testing and be linked to other phenotyping data only by anonymous Participant ID number. There is a rare risk of jaw and cheek tiredness, soreness, and pain, as well as dry mouth associated with producing a DNA saliva sample.

Failure to follow safety instructions of the study devices (Rare likelihood of risk)
Participants will be encouraged to review the Apple Watch or Fitbit User Guides before using Apple Watches or Fitbits for the study.

Risk from wearing the Apple Watch or Fitbit (Rare likelihood of risk)
A small number of people may experience reactions to certain materials. This can be due to allergies, environmental factors, extended exposure to irritants like soap or sweat, or other causes. Participants are advised to remove their Apple Watch or Fitbit and consult their physician if they experience redness, swelling, itchiness, or any other irritation.

Discomfort associated with being asked personal questions about health history and the completion of questionnaires (Infrequent likelihood of risk)
There is also the risk of psychological discomfort by study participants from questions asked in the assessments. Any participant becoming distressed while completing questionnaires will be encouraged to seek clarification of questions that they find to be unclear or troubling. The participant may skip any question that they find distressing. All participants are told that they have the option to terminate participation without penalty. Research staff will be trained to discuss any issues or concerns if they arise and will assist in arranging medical/ psychiatric help if necessary, including emergency treatment.

Discomfort associated with being asked questions about suicidal ideation and engaging in risk assessments (Likely risk)
There is the risk of psychological discomfort or stress by study participants surrounding questions being asked in the assessments about suicide and risk. Participants may experience some emotional discomfort or stress from discussing or answering these questions, including when being engaged in as-indicated risk assessments. The research staff conducting the assessments will be trained to respond to this emotional discomfort or stress and refer participants to appropriate resources as necessary, including supervisory backup within the research team. The Investigators or Project Manager will conduct face-to-face trainings with research staff to comprehensively review the Risk Management Protocol, risk factors for suicide, and techniques to distinguish low-, moderate-, and high-risk participants. In addition, the Investigators and Project Manager will monitor the progress of the study and quality of the assessments, and conduct additional ongoing in-person training and re-training as necessary over the course of the study. In order to ensure participant safety, there is the rare likelihood of the need for breach of confidentiality if research staff or investigators determine that a participant is at significant risk of suicidal or homicidal crisis or if there is mention of child or elder abuse.

Potential Benefits

The data collected from this study will help researchers further understand the risk factors that are associated with health trajectories. By utilizing machine learning and monitoring mobile technology, genomic, and environmental data, healthcare professionals can better understand key predictors of treatment response for patients with mental health conditions. These data can lead to changes in practice and, subsequently, improve the quality of patient care. Additionally, participants may see improvement in their mental health symptoms and overall well-being as a result of engaging in the app-based interventions. Access to mHealth interventions has the benefit of expanding access to treatment and may have wider effects on the population.

Participants will also receive an Apple Watch or Fitbit free of charge, and may keep these devices following their completion of the study. With these devices, participants can benefit from the health and fitness features to improve their well-being and overall quality of life.

Some participants will gain satisfaction from contributing to research about mental health conditions, which we hope will add to our understanding of disease etiology and potential interventions. This study also provides an opportunity to explore the use of integrated health systems, electronic medical records, and patient reported outcomes as tools for research to improve healthcare.

STUDY SUPPORT

The study staff will serve as the first contact point for any questions and technical issues encountered by the participant during the study. Support will be available to participants via email, phone, or scheduled in-person visits, as well as prepared supporting material (e.g., frequently asked questions within the study app). The study staff will address all study-related questions and will support participants in setting up their devices to meet study requirements. If participants have non-study related questions on using their Apple Watch or Fitbit, UM may refer them to Apple or Fitbit customer support. Participants will be recommended not to provide information on their participation in the study to customer support in order to minimize the risk of personnel identifying the study participant. If devices are broken, study staff may arrange a device swap with used or new equipment as available, or direct participants to Apple or Fitbit customer support. This study does not cover any costs associated with damages to the study devices except device shipment if swap is done via mail. Study staff will also support participants in making sure their devices are setup in a study-compliant manner, including but not limited to support to reinstall app(s) after a phone replacement or app deletion.

In the event that study staff become aware of study tasks that indicate psychiatric emergency or demonstrate critical laboratory values or pulse irregularities, study staff will respond pursuant to existing Michigan Medicine policies and generally accepted standards of care.

COSTS

There are no direct costs to the participant by participating in this study. All devices and the mobile applications are provided for free. There may be an indirect cost to participate in this study because some of the data collected for this study and transmitted to the study team may need to be covered by the participant's phone data plan.

If participants are found to have any medical or psychiatric condition, they are financially responsible for any follow-up treatment sought from their health care provider.

STUDY TERMINATION

The study may be terminated at any point. Participants will receive full compensation for their study task fulfillment up until the termination date. Participants will be informed of the study termination by phone, letter, or email and given contact information if they have questions for follow-up. Information will include directions on how to uninstall the study apps and reverse study-specific phone settings. Participants will no longer be contacted by study staff or receive notifications from the MyDataHelps study app. Participants will be informed of the possibility of early study termination during enrollment.

WITHDRAWING

Participants may withdraw from the study at any time, which will be communicated to participants by study staff and in the electronic informed consent form. Deleting the study app does not withdraw a participant from the study. Participants who choose to withdraw prior to completing the study will have no additional data collected, but data collected prior to the date of withdrawal can be used for research or product development projects initiated after the date of withdrawal. Alternatively, participants may opt for a complete withdrawal, which constitutes a request that their previously collected data not be used for future research or product development projects. Biological specimens will be destroyed and genomic and study data and medical information will not be available for new research or product development projects that are not already in progress. However, if the data are already in use for an existing research or product development project, such data may continue to be used for this project. This message is conveyed to all participants in the electronic informed consent process. Participants can withdraw by contacting study staff by phone or email, and study staff will initiate any necessary follow-up contact with participants by letter, phone, or email to confirm withdrawal. Contact will consist of follow-up on reason for withdrawal and information on how to uninstall the study apps and reverse study-specific phone settings. Participants will no longer be contacted by study staff or receive notifications from the MyDataHelps study app.

Participants may also be withdrawn from the study by the investigators for any of the following reasons:

a. Adverse event that renders the participant ineligible or makes it not in the best interest of the participant to continue participation in the opinion of the investigators
b. Observed protocol violation, including being observed to no longer meet inclusion criteria, a change that renders data less usable, or loss of follow-up
c. Observed to meet any of the exclusion criteria after enrollment
d. Death of participant

Participants will be informed of the withdrawal by phone, letter, or email and given contact information if they have questions for follow-up. Information will include instructions on how to uninstall the study apps and reverse study-specific phone settings. Participants will no longer be contacted by study staff or receive notifications from the MyDataHelps study app. As outlined in the electronic informed consent document, participants may contact study staff if they would like to opt for a complete withdrawal, as described above.

Collection of Participant Feedback

Throughout the project period, the study team may ask research participants to provide additional feedback about their experiences or a topic relevant to study activities and/or study aims. Participants may be asked to provide feedback through survey interviews or survey questionnaires. Participants will be selected to provide feedback by the study team, based on their level of study engagement. A study team member will transcribe interviews/participant responses while survey questionnaires will be sent and completed electronically through the study app (MyDataHelps). Prior to obtaining participant feedback, the study team will submit the questions for the survey interviews or questionnaires for IRB review and approval.

Table 1: Study Measures and Administration Time Points

Measure	Areas of Assessment	Administration Schedule
Demographics	Age, Sex, Race, Ethnicity, Education, Housing, Income	Baseline
Zip Code<br>(Current or Permanent Address)	Social Determinants of Health	Baseline
Risky Families Questionnaire	Early Family Environment	Baseline
NEO-Five Factor Inventory<br>(Select items - Neuroticism Items)	Neuroticism<br>(Domain of Personality)	Baseline
Pittsburgh Sleep Quality Index	Sleep Quality And Disturbance	Baseline
Mental Health Symptom Assessment	Mood Self-Report	Daily
Brief Suicide Risk Measure<br>(Czyz, et al., 2016)	Suicide Risk	Weekly
Mental Health App Assessment Questions -<br>(Adapted from Kern, et al., 2018)	Frequency of Use of Mental Health, Physical Activity, and Mindfulness Apps	Baseline<br>6 weeks<br>18 weeks<br>12 months
Patient Health Questionnaire (PHQ-9)	Depression	Baseline<br>6 weeks<br>18 weeks<br>12 months
Positive and Negative Suicide Ideation Inventory<br>(PANSI)	Suicide Risk Assessment	Baseline<br>6 weeks<br>18 weeks<br>12 months

Generalized Anxiety Disorder 7-Item Scale (GAD-7)	Anxiety	Baseline<br>6 weeks<br>18 weeks<br>12 months
Posttraumatic Stress Disorder Checklist (PCL-5) with PC-PTSD stem question	Post-Traumatic Stress Disorder	Baseline<br>6 weeks<br>18 weeks<br>12 months
Alcohol, Smoking, and Substance Involvement Screening Test (ASSIST)	Substance Use (excluding opioid use)	Baseline<br>6 weeks<br>18 weeks<br>12 months
Current Opioid Misuse Measure (COMM)	Opioid Misuse	Baseline<br>6 weeks<br>18 weeks<br>12 months
Interpersonal Support Evaluation List (ISEL-12)	Brief Social Support Inventory	Baseline<br>6 weeks<br>18 weeks<br>12 months
Satisfaction and Engagement with Care	Self-reported satisfaction with care and reasons for no-shows	18 weeks
Numerical Rating Pain Intensity Scale (NRS)	Pain	Baseline<br>6 weeks<br>18 weeks<br>12 months
