# Supplementary material for: Comparative Effectiveness of Three Digital Interventions for Adults Seeking Psychiatric Services: A Randomized Clinical Trial
Source: JAMA Netw Open. 2024 Jul 18;7(7):e2422115. doi: 10.1001/jamanetworkopen.2024.22115 (PMC11258584; doi:10.1001/jamanetworkopen.2024.22115)
Supplement: Supplement 2. — eFigure 1. Symptom-Level Change in Depressive Symptoms Across Intervention Arms eFigure 2. Symptom-Level Change in Anxiety and Suicidality Symptoms Across Intervention Arms [file jamanetwopen-e2422115-s002.pdf]

Supplemental Online Content

Horwitz AG, Mills ED, Sen S, Bohnert ASB. Comparative effectiveness of three digital interventions for adults seeking psychiatric services: a randomized clinical trial. *JAMA Netw Open*. 2024;7(7):e2422115. doi:10.1001/jamanetworkopen.2024.22115

eFigure 1. Symptom-Level Change in Depressive Symptoms Across Intervention Arms

eFigure 2. Symptom-Level Change in Anxiety and Suicidality Symptoms Across Intervention Arms

This supplemental material has been provided by the authors to give readers additional information about their work.

eFigure 1. Symptom-level change in depressive symptoms across intervention arms

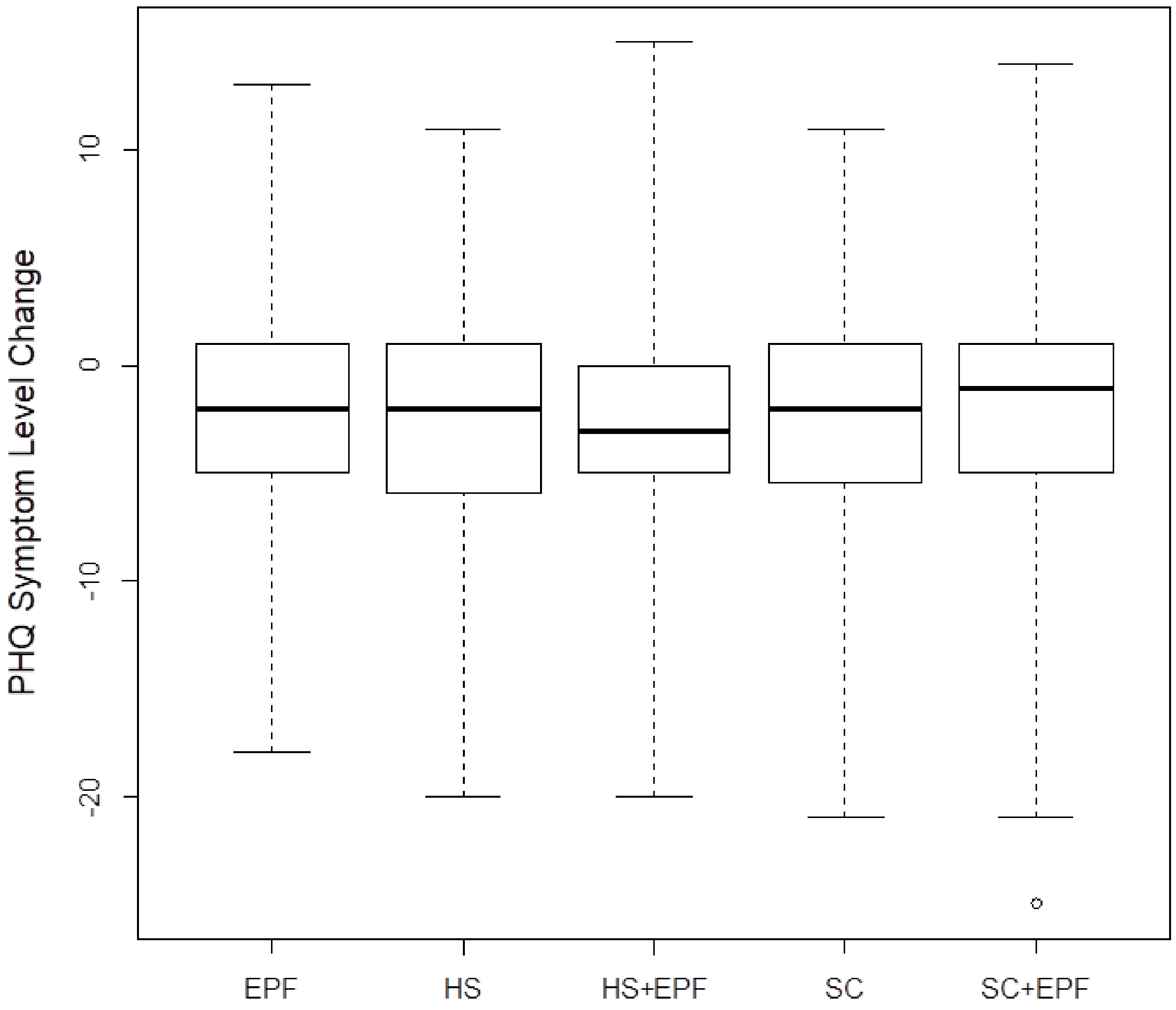

© 2024 Horwitz AG et al. *JAMA Network Open*

eFigure 2. Symptom-level change in anxiety and suicidality symptoms across intervention arms

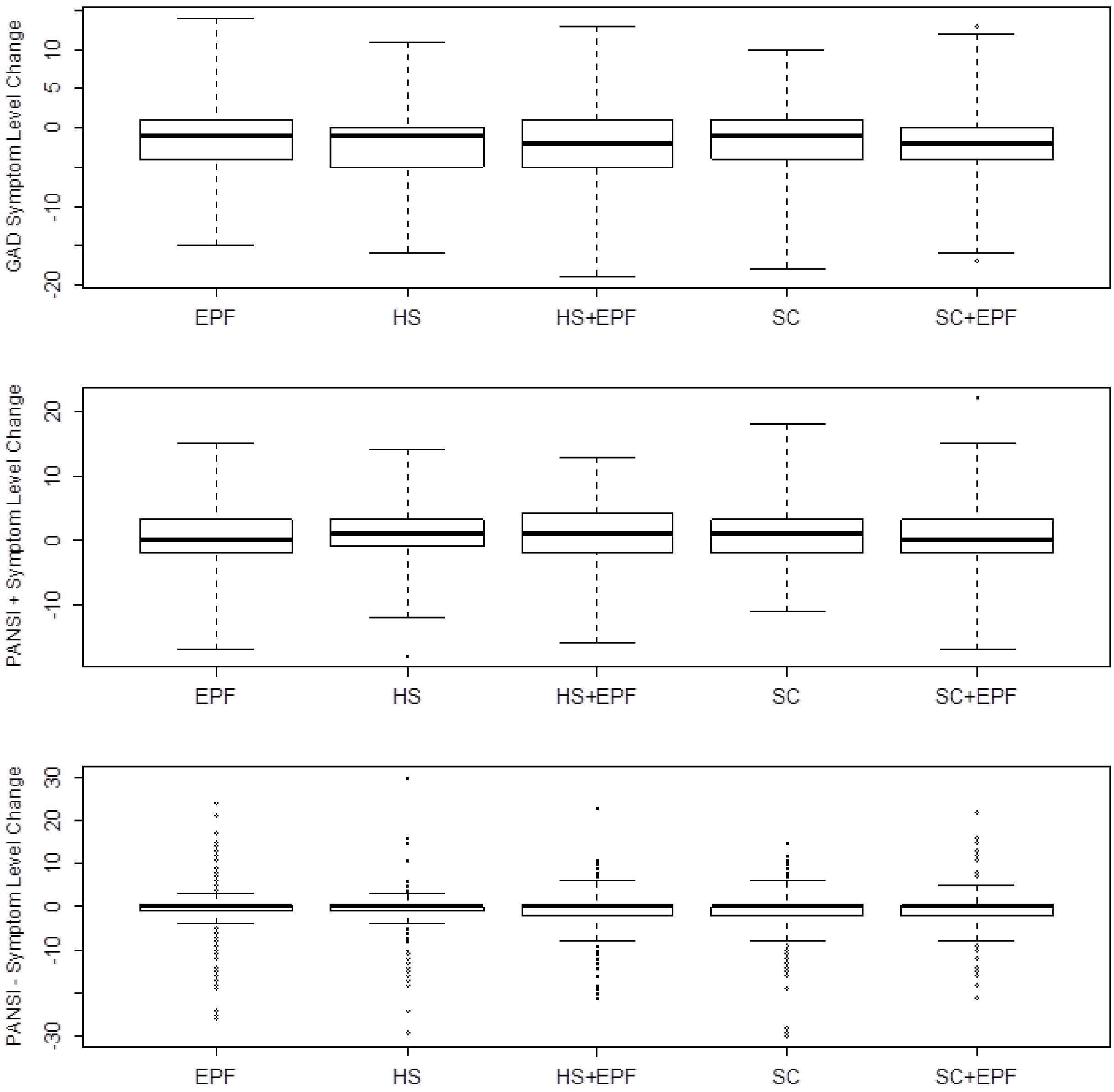

© 2024 Horwitz AG et al. *JAMA Network Open*
