# Supplementary material for: Comparative Effectiveness of Three Digital Interventions for Adults Seeking Psychiatric Services: A Randomized Clinical Trial
Source: JAMA Netw Open. 2024 Jul 18;7(7):e2422115. doi: 10.1001/jamanetworkopen.2024.22115 (PMC11258584; doi:10.1001/jamanetworkopen.2024.22115)
Supplement: Supplement 3. — Data Sharing Statement [file jamanetwopen-e2422115-s003.pdf]

Data Sharing Statement

Horwitz. Comparative Effectiveness of Three Digital Interventions for Adults Seeking Psychiatric Services. *JAMA Netw Open*. Published July 18, 2024.
doi:10.1001/jamanetworkopen.2024.22115

Data
Data available: Yes
Data types: Deidentified participant data, Data dictionary
How to access data: Data may be requested and accessed through the precision health analytics platform, at the following website: https://precisionhealth.umich.edu/tools-resources/data-access-tools/
When available: With publication

Supporting Documents
Document types: Other (please specify)
Additional Information: As a clinical trial, the trial protocol, including the statistical analysis plan, will be included as an online supplement
How to access documents: Specific requests for code or additional study documents can be sent to Dr. Amy Bohnert, amybohne@med.umich.edu.
When available: With publication

Additional Information
Who can access the data: Individuals may access the data through the Precision Health Analytics Platform, which does require specific criteria to be met in order to ensure data safety and patient privacy. Requirements can be viewed at this site:
https://precisionhealth.umich.edu/tools-resources/data-access-tools/how-to-access-data-tools-analytic-environments/
Types of analyses: Once the above criteria are met, investigators are able to analyze data according to the pre-specified purpose.
Mechanisms of data availability: Data will be made available when specified criteria are met (https://precisionhealth.umich.edu/tools-resources/data-access-tools/how-to-access-data-tools-analytic-environments/), no financial support is required.
